# Macular Hole: From Diagnosis to Therapy

**DOI:** 10.1155/2020/1473763

**Published:** 2020-03-14

**Authors:** Claudio Azzolini

**Affiliations:** Department of Medicine and Surgery, University of Insubria, Varese, Italy

Macular holes have attracted significant interest over the last few years due to the progress made in imaging and pharmacological and surgical therapies. The tractional interaction between foveal tissue and the vitreous has been extensively studied to identify vectorial forces, as well as the role of different cell subtypes in the pathogenesis and repair of retinal alterations [1]. At the same time, vitreoretinal surgery devices and instruments have advanced, increasing the safety, effectiveness, and repeatability of the surgical maneuvers [2].

This special issue encompasses cutting-edge research and review articles focusing on new perspectives on macular hole diagnosis and therapy. It includes novel research articles and reviews describing new insights into different types of macular holes, new instrumental OCT imaging, histopathology evaluations, and therapy. The main topics of the included manuscripts are summarized below to better classify and underline each topic.

In *idiopathic macular hole*, it is demonstrated that the dynamic forces caused by mobile posterior cortical vitreous with fluid currents exist already at early stages of macular hole development. A macular hole with a diameter of less than 250 *μ*m may have more chance of closing spontaneously. In addition, these spontaneously closed macular holes possess some distinctive OCT characteristics. Metamorphopsia is significantly related to the deterioration of visual quality of life and can be used as an independent index to evaluate visual function before and after surgery. A systematic literature search was conducted to identify and review studies comparing SF_6_ to C_3_F_8_ gases as a tamponade agent in the intraoperative management of macular holes, with no evidence found to support C_3_F_8_ gas as the tamponade agent of choice for macular hole surgery. Finally, several clinical studies evaluated different approaches applying the inverted flap technique for recurrent and large idiopathic as well as myopic macular holes [3–7].

In *lamellar macular hole*, a clinical study which evaluated the two different subtypes of LMH found peculiar functional aspects due to their morphological features; tractional lamellar macular holes revealed higher visual acuity and retinal sensibility due to the relative preservation of the outer retinal layers compared to degenerative lamellar macular holes. Another clinical study demonstrated that an accurate diagnosis of full thickness macular hole, lamellar macular hole, and myopic macular hole is important to determine the most appropriate surgical treatment of these lesions. Different options may be selected according to the OCT and fundus autofluorescence imaging findings [8].

In *traumatic macular hole*, a review on its etiology and management discusses about the role of tangential and anteroposterior vitreomacular traction. This macular hole may also result from foveal photoreceptor atrophy following commotio retinae. Spontaneous closure can occur; however, vitrectomy combined to the induction of posterior vitreous detachment and inner limiting membrane (ILM) peeling (with or without an ILM flap to cover the macular hole) is associated with anatomic success rates of up to 100%. Another research paper evaluated the outcome of surgery with and without an ILM flap, underlining the benefits of the ILM flap technique.

In *myopic macular hole*, an interesting review on surgical techniques underlines how myopic eyes still remain a challenge for vitreoretinal surgeons. Several and complex tractional mechanisms are implicated in the development of macular holes. The introduction of significant innovations for the treatment of myopic macular holes has allowed vitreoretinal surgeons to perform safe and effective surgery in these complicated eyes. Another research article on ILM peeling and inverted flap, which investigated the microstructural changes after successful myopic macular hole surgery, underlines that the inverted ILM flap technique did not affect the myopic macular hole healing processes compared to complete ILM removal [6]. Thus, the presence of the ILM plug did not interfere with the restoration of both external limiting membrane and ellipsoid zone, which correlated with functional recovery [5, 9].

In a research article on *the histology of epiretinal proliferative tissue*, the authors investigated the morphological characteristics and cell composition of various types of surgically excised proliferative membranes and internal limiting membranes. Cell components such as myofibroblasts, macrophages, and polymorphonuclear cells were recognized as the expression of cell migration and differentiation that induced an inflammatory process and a fibroproliferative repair process. The detection of pigments in specific types of epiretinal membrane suggested that retinal pigment epithelium cells might have a role in the development of these vitreoretinal disorders.

In a review evaluating *new experimental approaches in autologous cell transplantation as a new therapy*, the application of mesenchymal stem cells (MSC) described an adjuvant treatment for refractory and late-stage macular holes to be considered a promising direction. The combination of pars plana vitrectomy and MSC injections or MSC exosome application appears feasible, but the evidence regarding their safety needs to be expanded. Long-term clinical trials featuring control groups are still required in order to compare the efficacy of MSC applications with pars plana vitrectomy and other adjuvant techniques.

In summary, this special issue offers an overview of different types of macular holes, as well as providing new findings that may offer directions for new research in this important field.
